# The Natural Product Citral Can Cause Significant Damage to the Hyphal Cell Walls of *Magnaporthe grisea*

**DOI:** 10.3390/molecules190710279

**Published:** 2014-07-15

**Authors:** Rong-Yu Li, Xiao-Mao Wu, Xian-Hui Yin, Jing-Nan Liang, Ming Li

**Affiliations:** 1 Institute of Crop Protection, Guizhou University, Guiyang 550025, China; 2 The Provincial Key Laboratory for Agricultural Pest Management in Mountainous Region, Guizhou University, Guiyang 550025, China; 3 Key Laboratory of Green Pesticide and Agricultural Bioengineering, Ministry of Education of China, Research and Development Center for Fine Chemicals, Guizhou University, Guiyang 550025, China; 4 Instrument Center of Institute of Microbiology Chinese Academy of Sciences, Institute of Microbiology, Chinese Academy of Science, Beijing 100101, China

**Keywords:** *Magnaporthe grisea*, antifungal activity, natural product citral, electron microscopy, chitinases

## Abstract

In order to find a natural alternative to the synthetic fungicides currently used against the devastating rice blast fungus, *Magnaporthe grisea*, this study explored the antifungal potential of citral and its mechanism of action. It was found that citral not only inhibited hyphal growth of *M. grisea*, but also caused a series of marked hyphal morphological and structural alterations. Specifically, citral was tested for antifungal activity against *M. grisea in vitro* and was found to significantly inhibit colony development and mycelial growth with IC_50_ and IC_90_ values of 40.71 and 203.75 μg/mL, respectively. Furthermore, citral reduced spore germination and germ tube length in a concentration-dependent manner. Following exposure to citral, the hyphal cell surface became wrinkled with folds and cell breakage that were observed under scanning electron microscopy (SEM). There was damage to hyphal cell walls and membrane structures, loss of villous-like material outside of the cell wall, thinning of the cell wall, and discontinuities formed in the cell membrane following treatment based on transmission electron microscopy (TEM). This increase in chitinase activity both supports the morphological changes seen in the hyphae, and also suggests a mechanism of action. In conclusion, citral has strong antifungal properties, and treatment with this compound is capable of causing significant damage to the hyphal cell walls of *M. grisea*.

## 1. Introduction

Rice (*Oryza sativa*) is one of the most important food crops in the world, according to the International Rice Research Institute database, and is cultivated worldwide on over 153 million hectares [1]. However, rice blast disease (RBD), caused by the fungus *Magnaporthe grisea*, is a significant and recurring problem in China and other rice-growing regions [2,3]. The fungus is a filamentous ascomycete with a broad host range and worldwide distribution. It is one of the most devastating of all cereal diseases due to the critically decrease in both rice yield and quality [1,4], and it causes between 10%–30% of the annual rice harvest losses [2,5].

One common solution to controlling these pathogenic fungi is the use of synthetic chemical fungicides, however, their use often results in other problems. These problems include threatening human health and the environment by supporting the emergence of resistant pathogens [3,6,7,8], and by leaving pesticide deposits on food [6]. Therefore, naturally occurring plant products are important sources of antifungal compounds with low toxicity to mammals and safe to the environment which may serve as substitutes for synthetically produced fungicides [9,10]. Compared to synthetic chemical fungicides that show insecticidal activity, botanical fungicides are recognized to have relatively low or little toxicity [11], but they can play an important role in reducing environmental pollution in agricultural ecosystems [12]. 

A key component of the essential oils from *Litsea cubeba* is citral (3,7-dimethyl-2-7-octadienal), which is extracted from the *L. cubeba* fruits. Citral is an acyclic α,β-unsaturated monoterpene aldehyde, and an isoprenoid compound made up of the isomers geranial and neral [13,14,15], Because of its high bioreactivity, including antimicrobial (human pathogenic fungi and bacterium) and insecticidal properties, citral has been the focus of attention of numerous researchers [16,17,18]. However, the application of natural compound citral against fungal diseases for agriculture is very minor, like other plants extracts which are effective, selective, biodegradable, and less toxic to the environment. In addition, there is little study related to the investigation of compounds which are responsible for the bioactivities of citral, and its exact mechanism of antimicrobial action. Despite the previous research on citral, its effect on *M. grisea* and corresponding mechanism of action have not yet been elucidated. Therefore, the goal of the present study was to investigate the antifungal activity of citral isolated from *L. cubeba* oils on *M. grisea,* and to describe the effects on the gross morphology and ultrastructure of *M. grisea* hyphae by scanning electron microscopy (SEM) and transmission electron microscopy (TEM). 

## 2. Results and Discussion

### 2.1. Effects of Citral on Fungal Hyphal Growth

**Figure 1:**
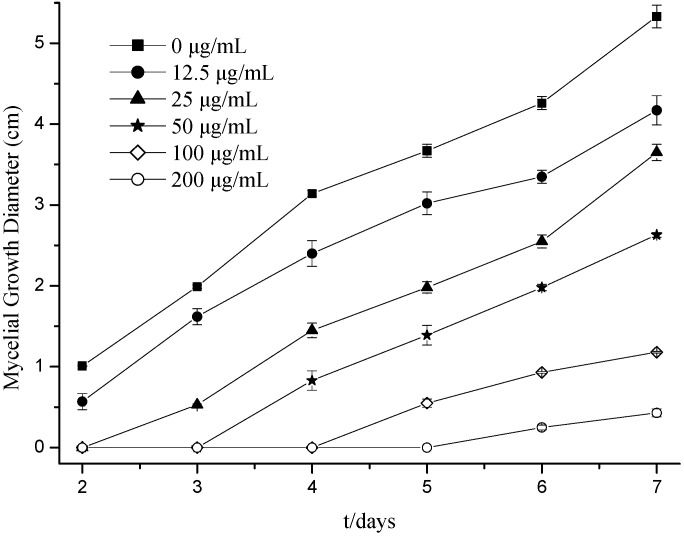
Effect of citral on *M. grisea* mycelial growth.

The effect of citral on mycelial growth of *M. grisea* was determined for a range of citral concentrations as shown in Figure 1. It was found that supplementing with citral significantly reduced *M.grisea* colony development on potato dextrose agar (PDA) 6 days after inoculating. Specifically, a citral concentration of 200 μg/mL inhibited growth to 0.25 cm, and 12.50 μg/mL up to 4.26 cm. Moreover, the highest citral concentration tested, 200 μg/mL, resulted in complete (100%) inhibition of fungal colony development at 2–5 days, after which mycelial growth recommenced. Following a 7 days inoculation, the antifungal activity of citral was 21.76% at 12.50 μg/mL and 91.93% at 200 μg/mL. Furthermore, citral significantly inhibited the mycelial growth of *M. grisea* in a concentration-dependent manner with IC_50_ and IC_90_ values of 40.71 and 203.75 μg/mL, respectively. A previous study reported that citral at concentrations of 50 and 100 μg/mL can decrease *Trichophyton mentagrophytes* growth in PDA by 8.5% and 100%, respectively [19]. These results also agree with those of Saddiq and Khayyat [17] and Wuryatmo *et al.* [20], who reported on the strong antifungal activity of citral.

### 2.2. Effects of Citral on Germination of Fungal Spores

The effect of citral on *M. grisea* conidia germination and germ tube elongation were measured and the results are listed in Table 1. It was found that spore germination was significantly inhibited by citral in a concentration-dependent manner, and a similar trend was observed for mycelial growth. When treating with 200 μg/mL citral, there was a 5.20% inhibition of spore germination. Overall, the IC_50_ of citral was 38.81 μg/mL. Spore germination increased as the citral concentration was reduced, which resulted in an increase in *M. grisea* germ tube length. As the citral concentration went from 200 μg/mL to 12.50 μg/mL, the germ tube length of *M. grisea* went from 12.67 μm to 175.67 μm, respectively. Conidia germination and appressorium formation were also suppressed by citral treatment of ≥400 μg/mL. These results confirm previously published work, such as that by Silva *et al.* [16], and Ganjewala *et al.* [21], Daferera *et al.* [22], concerning the efficacy of citral for inhibition of pathogenic fungi growth.

**Table 1:** Effect of citral on the spore germination and germ tube length of *M. grisea*.

Citral Treatment(μg/mL)	Spore Germination Rate (%)	Germ Tube Length (μm)
0	100.00 a	175.67 a
12.5	89.66 b	123.78 b
25	70.90 c	91.11 c
50	34.47 d	54.44 d
100	14.40 e	30.11 e
200	5.20 f	12.67 f
400	0.00 j	0.00 j

Values represent means of measurements for six independent plates per treatment. In each column, the values followed by the same letter do not differ significantly for *p* < 0.05.

The described inhibitory activity of citral against fungi is most likely due to citral being a member of the α,β-unsaturated aldehyde class in which the carbonyl group is adjacent to the α and β-carbons. Because of their location on the molecule, the α and β-carbons are conjugated with the carbonyl group, which makes the α-carbon positively polarized and able to react easily with nucleophiles and undergo nuclephilic attack [23]. According to Witz, the chemical nature of α,β-unsaturated aldehydes, as well as some of their toxicological effects, are based on their ability to function as direct alkylating agents [24]. These alkylating agents are capable of binding covalently to cellular nucleophile groups, meaning they are capable of modifying cellular processes and have potential toxicity.

### 2.3. Electron Microscope Analysis of M. grisea

**Figure 2:**
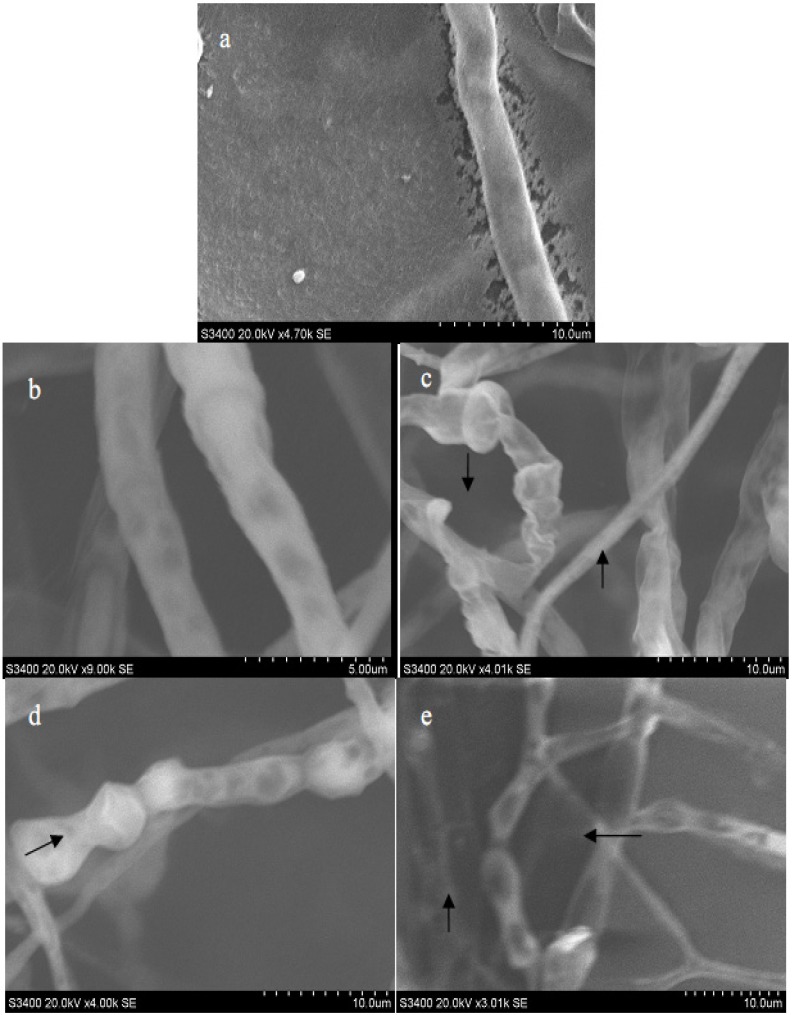
Scanning electron micrographs of *M. grisea*: Hyphae exposed to citral at concentrations of (**a**) 0 μg/mL, (**b**) 50.00 μg/mL, (**c**) 100.00 μg/mL, (**d**) 150.00 μg/mL, and (**e**) 200.00 μg/mL. Arrows and arrowheads indicate hyphae shrinkage and partial distortion, respectively.

Observations made by SEM indicated that untreated *M. grisea* hyphae had a complete tubular shape and were morphologically normal (Figure 2a). After exposure to a citral concentration of 50.00 μg/mL, the hyphae still maintained an almost normal morphology (Figure 2b). However, once the concentrations of citral were increased to 100.00, 150.00 and 200.00 μg/mL, the hyphae displayed aberrant morphologies, including shrinkage, partial distortion and wrinkling of the surface (Figure 2). Such morphological changes were also evident in the hyphae treated with 100.00 μg/mL citral (Figure 2c). These distorted hyphae had a flattened appearance. Similar conclusions have also been made in previous studies by Maoz and Neeman [25], and Xiong *et al.* [26], who also found that hyphae morphology becomes irregular under certain treatment conditions. 

**Figure 3:**
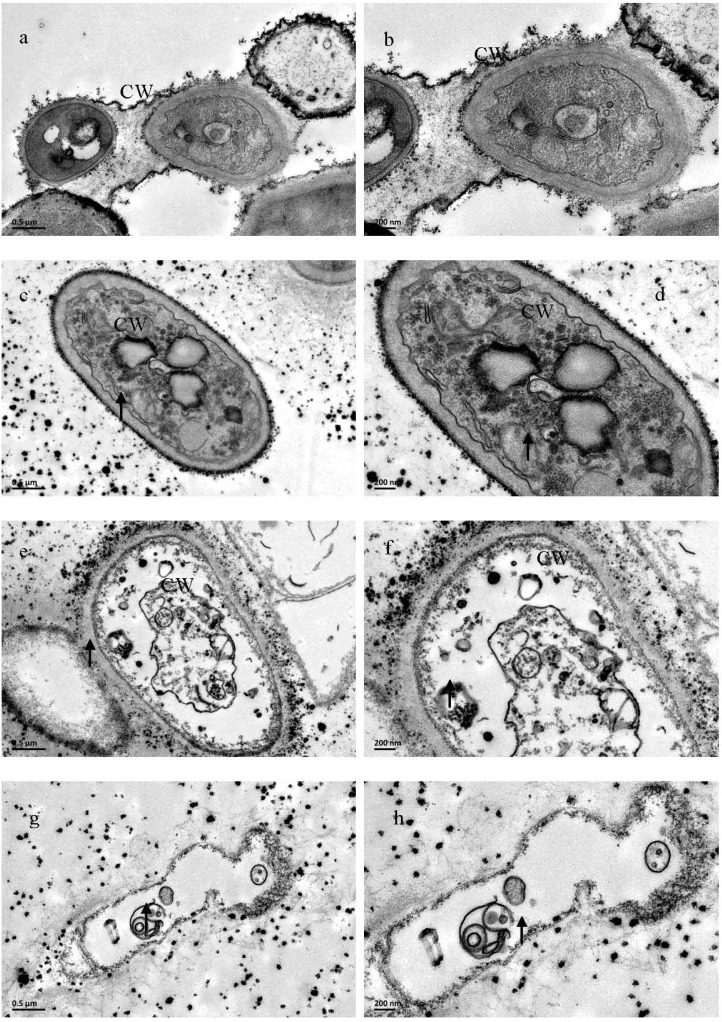
Transmission electron micrographs of *M. grisea* hyphae, where hyphae was exposed to agar with citral at (**a**, **b**) 0 μg/mL, (**c**, **d**) 50.00 μg/mL, (**e**, **f**) 100.00 μg/mL, and (**g**, **h**) 200.00 μg/mL. CW: Cell wall.

TEM studies of untreated *M. grisea* hyphae revealed the presence of typical eukaryotic cytoplasmic components, including numerous ribosomes, mitochondria and vacuoles in the cytoplasm enclosed by an electron-transparent cell wall (Figure 3a,b). The mitochondria had well-developed cristae that projected into the matrices. On the other hand, mycelia that had been treated with citral showed ultrastructural changes in the hyphal morphology. Following exposure to 50.00 μg/mL citral, the cell walls of the hyphae became rough, and some vesicles containing electron dense structures appeared in the vacuoles (Figure 3c,d). When hyphae were exposed to 100.00 μg/mL citral, the cellular degeneration was severe, with the presence of indiscernible organelles in the cytoplasm, and cell ghosts were often found where various round shaped structures appeared. Furthermore, the cell walls appeared to be degraded and had an exfoliated texture (Figure 3e,f). After increasing the concentration of citral to 200.00 μg/mL, the cytoplasm almost completely disappeared and the cell wall ruptured. There was also a remarkable increase in the number of cell ghosts (Figure 3g,h). Overall, the treated cells became abnormal and displayed cellular disorganization upon exposure to citral, suggesting that citral ruptures the cell wall, penetrates the cell membrane and interacts with cellular organelles. These phenomena that were observed agree with Arlorio *et al.* [27], who observed the rupture of fungal cell walls upon exposure to citral. These phenomena are likely due to the structure of citral, because the effects of solutes on cell walls might be influenced by compounds that possess a hydrophilic group and an electron acceptor group, such as an aldehyde group [25,26].

### 2.4. Effect of Citral on Chitinases Activity of M. grisea

**Figure 4:**
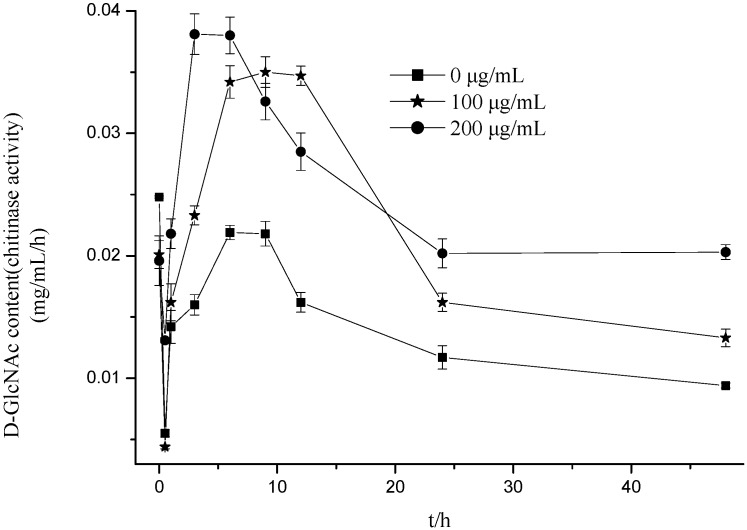
Effect of citral on the chitinase activity of *M. grisea*.

Chitinases are digestive enzymes that break down glycosidic bonds in chitin. In fungi, chitinase has been to be shown to be involved in regulating degradation of cell walls, germination of spores, tip growth, branching of hyphae, detachment of spores, and autolysis of hyphae [28,29,30]. The present paper demonstrated the effect of citral on the chitinase activity of *M. grisea* (Figure 4). Treatment with citral significantly increased the activity of the enzyme in the sample as compared to the control. Within the first 3 h of citral treatment, the chitinase activity of *M. grisea* decreased, and then after 3 h, the activity increased. Six h after the treatment, the chitinases displayed the highest level of activity at the highest citral concentration of 200 μg/mL with an activity increase of 73.52% as compared with the blank group. When treating with the concentration of 100 μg/mL, the chitinase activity had increased 60.55% as compared with the control 9 h after treatment. By 24 h after treatment, chitinase activity was stable. Therefore, citral can significantly increase chitinase activity, and thereby cause degradation of cell walls. This results fits with the the morphological changes in the hyphae with citral treatment, and agrees with the results of Maoz and Neeman [25], Adams [30], Mauch *et al.* [31] Sela-Buurlage *et al*. [32], and Bruno and Donzelli [33], who have also observed that chitinase is involved in the regulation of cell wall degradation. 

## 3. Experimental Section

### 3.1. Chemicals and Samples

*M*. *grisea* strain ZB15 was isolated from a diseased rice panicle taken from Guizhou Province in China and incubated on potato dextrose agar (PDA) plates at 28 °C. A single-spore isolate was obtained and maintained at 4 °C on PDA. Citral (purity ≥ 95%) was isolated from *Litsea cubeba* essential oils, dissolved in acetone to make a 1 mg/mL stock solution, and stored at 4 °C. PDA or potato dextrose broth (PDB) was used for routine cultures. Rice bran medium (RBM) was prepared for conidia propagation by boiling 20.0 g of rice brain, adding 10.0 g each of sucrose and agar, and diluting to a final volume of 1 L with distilled water.

### 3.2. Impact of Citral on Fungal Hyphal Growth

The antifungal tests of citral was carried out for assessing the effects towards mycelial growth of *M. grisea* as described previously [34]. For determination of contact effects, citral was dispersed as an emulsion in water using dimethyl sulfoxide (DMSO) and Tween 80 (0.1% v/v) and added to PDA immediately before it was emptied into the glass Petri dishes (90 mm in diameter) at a temperature of 40–45 °C. The concentrations tested were 12.5 to 200 μg/mL. The controls received the same quantity of DMSO and Tween 80 mixed with PDA. Then the plates were kept under UV light in the laminar flow chamber for solidification of the media. After solidification, a 6 mm diameter disc cut from the actively growing front of a 1-week old colony of the desired pathogenic fungus was then placed with the inoculum side down in the center of each treatment plate, aseptically *.* Treated petri dishes were then incubated at 28 °C till the fungal growth was almost complete in the control plates. All experiments were in quadruplet for each treatment against *M. grisea*. The diameters of the fungal growth were measured after 2, 3, 4, 5, 6 and 7 days. The formula for calculating the growth inhibition of fungal hyphae was as follows:

Inhibition (%) = (1 − Dt/Dc) × 100
(1)

where Dt and Dc were the growth zone diameters in the experimental dish (mm) and the control dish (mm), respectively. Concentrations of compounds that inhibited 50% of the mycelium growth (IC_50_ values) were calculated by probit analysis [34].

### 3.3. Impact of Citral on Fungal Spore Germination

In order to determine the effect citral has on fungal spore germination, the following procedure was performed. PDA in Petri dishes were inoculated with mycelial plugs of *M. grisea* and incubated at 28 °C. After 14 days, conidia of *M. grisea* in each dish were harvested and air dried at room temperature. These *M. grisea* conidia were submerged in 20 mL of citral solutions at a concentration of 12.5, 25, 50, 100, 200, and 400 μg/mL at 28 °C for 24 h or submerged in distilled water. The conidia were rinsed three times for 1 min each in sterile distilled water. Spore germination and morphological changes were monitored microscopically. The percentage of germinated conidia that formed appressoria was determined by direct microscopic examination of at least 100 spores per replicate [6]. The inhibition of spore germination was calculated from the experimental mean values as follows:

Inhibition (%) = 100 × (*A* – *B*)/*A* (2)

where *A* and *B* are the percentages of germinated spores in the control and test sample, respectively. The IC_50_ value was calculated by probit analysis.

### 3.4. Morphological Study

For morphological studies, treatments were prepared by adding different concentrations of citral in PDA to each petri dish, and then growing *M. grisea* on these Petri dishes at 28 °C for 10 days. Samples for SEM observation were prepared as follows: mycelia were obtained after exposure to citral and then fixed with 4% (v/v) glutaraldehyde at 4 °C overnight. After fixing, mycelia were washed briefly with 0.1 mol/L phosphate buffered saline (PBS, pH = 7.2) twice, and then dehydrated in a graded t-butanol series of 30%, 50%, 70%, 90% and then 100% three times for 10 min each. The dehydrated specimens were dried in a freeze drier (LGJ-10D; Beijing Fourth Ring Scientific Instrument Co., Ltd., Beijing, China), and sputter-coated with gold. Observations were carried out using a SEM (S-3400N; Hitachi, Tokyo, Japan) operated at an accelerating voltage of 20 kV. Untreated mycelia were processed alongside test samples as a control.

For TEM analysis, mycelia samples were processed as follows. The specimens were fixed with 2.5% (v/v) glutaraldehyde and 2.0% (v/v) paraformaldehyde in 0.1 mol/L PBS (pH = 7.2) at 4 °C overnight, and then washed with PBS three times for 15 min each. The specimens were further fixed with 2% potassium permanganate (KMnO_4_) for 2 h, washed with PBS three times for 15 min each, dehydrated in a graded acetone series of 30%, 50%, 70%, 85%, 95% and then 100% for 10 min each, and embedded in Spi-pon-812 resin. Ultrathin sections approximately 60 nm thick were made by an ultramicrotome (UC-7; Lecia Inc., Wetzslar, Germany) with a diamond knife. Ultra-thin sections were stained with uranyl acetate then lead citrate, and viewed on a TEM (JEL-1400; JEOL Ltd., Tokyo, Japan). As a control, untreated mycelia were also processed for TEM. 

### 3.5. Impact of Citral on Chitinases of M. grisea

The chitinase activity of *M. grisea* was measured using a previously published spectrophotometric method [35,36]. First, enzyme liquid extraction was performed by centrifuging 30 mL of treated mycelial suspension at 6,000 rpm for 15 min at 4 °C, and discarding the supernatant. The resulting pellet was mixed with approximately 4 mL of Tris-HCl buffer (pH = 7.5, 4 °C) and ground with a mortar and pestle into a slurry. The slurry was centrifuged at 10,000 rpm for 10 min at 4 °C. The supernatant was transferred into a 1.5 mL centrifuge tube and then stored at a low temperature until use. Second, enzyme activity was measured [37]. A solution was made by mixing 0.4 mL of the colloidal chitin suspension with 0.6 mL enzyme sample in a test tube, according to previous work by Boller *et al.* [38], and Nitoda *et al.* [35]. This solution was placed in a water bath at 37 °C for 60 min, while stirring with a magnetic stir bar. The solution was then boiled for 5 min to terminate the reaction, centrifuged at 10,000 rpm for 10 min, and the resulting supernatant was transferred into a 1.5 mL centrifuge tube. Next, 0.4 mL of supernatant fluid, 0.2 mL of K_2_B_4_O_7_ (0.8 mol/L), and 3 mL of 1% *p*-dimethylaminobenzaldehyde (DMAB) were mixed sequentially. This solution was incubated for 20 min at 36 °C, and then measured at 544 nm. One unit of enzyme was defined as the change in the amount of N-acetylglucosamine caused by chitinase enzymatic hydrolysis of chitin per hour. 

### 3.6. Statistical Analysis

All analyses were performed in four replicates. The data were analyzed using SPSS 12.0 statistical software package (SPSS, Inc., Chicago, IL, USA) and Microsoft Office Excel 2010. Data were first tested for normality and then analyzed by ANOVA. Significant differences between the mean values of each cohort were determined using Duncan's Multiple Range test (*p* < 0.05) following one-way ANOVA.

## 4. Conclusions

In conclusion, citral significantly inhibits the mycelial growth, conidial formation and germination, and appressorium formation of *M. grisea*. The mechanism of action of citral may be capable of causing significant damage to the hyphal cell walls of *M. grisea*, so the antifungal and toxicological characteristics of citral indicate its great potential for agricultural applications in the treatment of fungal pathogen infections, e.g., RBD caused by *M. grisea*. Further studies on citral, such as *in vivo* toxicological characteristics, phytotoxicity tests, and field experiments, are required to determine its potential as a commercial fungicide.
